# A Cellular Model of Infection with *Brucella melitensis* in Ovine Macrophages: Novel Insights for Intracellular Bacterial Detection

**DOI:** 10.3390/vetsci6030071

**Published:** 2019-09-03

**Authors:** Garyfalia Karponi, Spyridon K. Kritas, Eleni Papanikolaou, Evanthia Petridou

**Affiliations:** 1 Department of Microbiology and Infectious Diseases, School of Veterinary Medicine, Faculty of Health Sciences, Aristotle University of Thessaloniki, 54124 Thessaloniki, Greece (S.K.K.) (E.P.); 2 Laboratory of Biology, School of Medicine, National and Kapodistrian University of Athens, 11527 Athens, Greece

**Keywords:** brucellosis, macrophages, ruminant animals, infectious diseases

## Abstract

Intracellular bacteria provoking zoonoses, such as those of the genus *Brucella*, present a host cell tropism mostly limited to the monocyte/macrophage lineage, leading to chronic inflammatory reactions, difficult-to-eradicate-infections, and widespread prevalence among ruminants. Eradication of brucellosis has been based on programs that translate into a substantial financial burden for both the authorities and stockbreeders, if not strictly followed. To this end, we sought to create an in vitro cell model that could be utilized as future reference for adequately measuring the number of engulfed brucellae/cell, using peripheral blood-derived sheep macrophages infected with *B. melitensis* at decimal multiplicities of infection (MOI = 5000-5), to simulate the host cell/microorganism interaction and monitor bacterial loads up to 6 days post-infection. We show that the MOI = 5000 leads to high numbers of engulfed bacteria without affecting macrophages’ viability and that the minimum detection limit of our Real-Time PCR assay was 3.97 ± 5.58 brucellae/cell. Moreover, we observed a time-associated, significant gradual reduction in bacterial loads from Day 2 to Day 6 post-infection (*p* = 0.0013), as part of the natural bactericidal properties of macrophages. Overall, the work presented here constitutes a reliable in vitro cell model of *Brucella melitensis* for research purposes that can be utilized to adequately measure the number of engulfed brucellae/cell and provides insights towards future utilization of molecular biology-based methods for detection of *Brucella*.

## 1. Introduction

Intracellular infectious agents, such as bacteria of the genus *Brucella*, provoke abortions and infertility in ruminant animals [1] and present a host cell tropism mostly limited to the monocyte/macrophage lineage [2]. Thus, brucellosis is difficult to eradicate, since the microorganism may effectively evade antibiotic treatment. Currently, vaccination programs in livestock, monitoring, and slaughtering of seropositive carriers constitute the only available means for eradicating the disease [3]. However, the available animal vaccine [4] is sometimes ineffective [5] and even infectious for veterinarians [5]. Moreover, the disease cannot be prevented in humans, since no vaccine currently exists and antibiotic therapy is long-lasting, potentially leading to relapse or re-infection after de novo exposure to the microorganism [6]. Consequently, *Brucella* infection in livestock not only poses severe public health issues, but also translates into a substantial financial burden for stockbreeders [7]. Therefore the need to adequately monitor *Brucella* infection in flocks is necessary. Current methods for *Brucella* detection involve classic microbiological assays, i.e., colony-forming units (CFU) detection on agar plates as well as several serological assays based on immune response [8]. Surprisingly, molecular-based methods such as Real-Time PCR and classic end-point PCR are not considered effective in terms of surveillance of the prevalence in herds or flocks [8].

In an effort to understand the potential reasons that hamper the wider use of Real-Time PCR to detect *Brucella* infection, we aimed to generate an ovine macrophage infection model, using decimally diluted *B. melitensis*, to imitate the host cell/microorganism interaction in vitro and monitor the number of incorporated brucellae/cell by simultaneously comparing the classic CFU detection method with a molecular-based Real-Time PCR detection method. Our results indicate that the Real-Time PCR method has a lower sensitivity level of detection and suggest that potential future diagnosis for *Brucella* infection by Real-Time PCR can be reliable only if large numbers of infected cells are present.

## 2. Materials and Methods

### 2.1. Bacteria and Ovine Macrophages

*B. melitensis* 16 M strain (ATCC 23456) was purchased from Culture Collections Public Health England (Salisbury, UK). To store-purchased *B. melitensis* long-term, a standard procedure was followed, as per the manufacturer’s instructions, in order to open the freeze-dried vial containing the 16 M strain upon arrival. Then the strain was cultured aerobically both on *Brucella* agar (Oxoid, Hampshire, UK) and Columbia agar sheep blood plates (Oxoid, Hampshire, UK) for 3 days at 37 °C. From the initial culture, ten vials containing 10^8^ CFU/mL *B. melitensis* in brain heart broth (Oxoid, Hampshire, UK) with 15% glycerol (Sigma-Aldrich, St. Louis, MO, USA) were primarily screened for contamination and then stored at −80 °C, under the label “master seed”. Working seed was prepared by spreading 10 μL of a master seed vial on Columbia agar sheep blood plates. After 3 days of incubation, ten vials of working seed were prepared following the same protocol for the master seed. Also, initial titration of bacteria, was conducted utilizing a suspension turbidity detector (Liofilchem, Teramo, Italy). Purity of culture to *B. melitensis* was assessed by spreading working seed brucellae on Columbia agar sheep blood plates. To infect macrophages at particular MOIs, serial dilutions of cultured bacteria were plated on Columbia agar sheep blood plates (Oxoid, Hampshire, UK), and bacterial colony-forming units (CFU) were determined after 3 days to estimate bacterial concentrations per mL.

Ovine macrophages were obtained by culturing the mononuclear cell fraction of sheep peripheral blood (*n* = 6) after ficoll density gradient centrifugation. Characterization of the extracted and cultured primary macrophages was extensively described with flow cytometry and a phagocytosis assay as previously described [9]. 

### 2.2. Infection of Macrophages with B. melitensis

**Figure 1:**
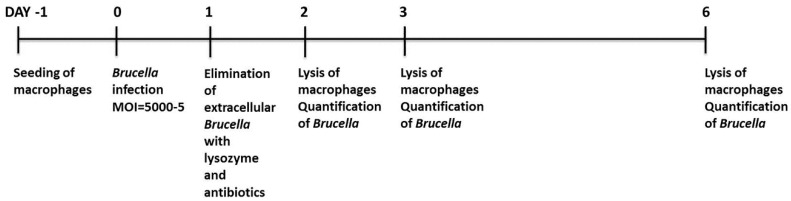
Schematic diagram and timeline of the study.

Fifty thousand ovine macrophages were seeded in 24-well tissue culture plates at a final volume of 1 mL (Day −1). 24 h later (Day 0), the cells were counted by trypan blue exclusion, washed with phosphate-buffered saline (PBS) (Lonza, Basel, Switzerland), and exposed to *Brucella* at an MOI of 5000, 500, 50, or 5. The macrophage-specific medium (Macrophage-SFM, Invitrogen, Carlsbad, CA, USA, supplemented with heat-inactivated fetal bovine serum, Invitrogen, Carlsbad, CA, USA) used for the duration of the whole cell culture, did not contain any antibiotics at this point. The next day (Day 1), cells were washed with PBS and cultivated in macrophage-specific culture medium supplemented with 5% penicillin/streptomycin (Invitrogen, Carlsbad, CA, USA) and 0.5 mg/mL lysozyme (Roche, Basel, Switzerland) to kill extracellular bacteria overnight. At the next day of the procedure (Day 2), cells were either washed with PBS to remove lysozyme, counted, and left in culture up to 4 more days (Day 3 or Day 6) in the macrophage-specific culture medium, or washed with PBS, detached from the culture plates with 0.05% trypsin-EDTA (Invitrogen, Carlsbad, CA, USA) and pelleted at 1400 rpm for 10 min, to exclude precipitating any extracellular brucellae that may had survived exposure to antibiotics and lysozyme. The pellets were resuspended in PBS and underwent 3 freezing-thawing cycles. Before DNA extraction, all samples were additionally incubated with 0.25 mg/mL lysozyme for 1 h at 37 °C. Quantification of internalized brucellae was conducted with quantitative Real-time PCR and CFU assay. Uninfected cells served as control at all times. Viability of macrophages was maintained by changing the cell culture medium and assessed by trypan blue exclusion at all time points of the experimental procedure. Especially for the time gap between Days 3 and 6, the wells were supplemented with the appropriate fresh medium volume in order to maintain the culture supernatant at 1 mL. The study is graphically outlined in Figure 1. 

At all time points, the presence or absence of live brucellae in the culture supernatant was verified by collecting the old culture media, spinning at 14,000 rpm and seeding the resuspended pellets in Columbia agar sheep blood plates. The same procedure was also applied in the supernatant collected from the final cell wash, that was implemented to remove trypsin, after harvesting the infected cells. To assess intracellular live brucellae, 500 infected cells were isolated and processed by adding 0.1% saponin (Sigma-Aldrich, St. Louis, MO, USA), incubated at room temperature for 5 min, pelleted at 14,000 rpm, resuspended in PBS and plated in Columbia agar sheep blood plates as previously described [10].

### 2.3. PCR Analysis of Brucella in Macrophages

Genomic DNA from cultured cells was extracted using the High Pure PCR template preparation kit (Roche, Basel, Switzerland) as per the manufacturer’s instructions. Furthermore, to exclude the presence of PCR inhibitors at a high concentration, we spiked an exogenous source of DNA template to our samples (Primerdesign, Chandler’s Ford, UK), which, after co-purification with our target DNA, was used as a positive control for the extraction process with Real-Time PCR.

Quantification of internalized brucellae, was conducted with the *Brucella* genus Genesig advanced kit (Primerdesign, Chandler’s Ford, UK). From initial experimentation, we realized that utilization of the kit’s standards leads to different quantification of brucellae. Therefore, we generated in-house standards based on various amounts of *B. melitensis* 16 M genomic DNA deriving from lysed brucellae cells cultivated in our laboratory for the detection of the L-glutamine: 2-deoxy-scylloinosose aminotransferase gene as indicated by the commercial kit. Based on these results the number of brucellae that can be quantified ranges from 2.5 × 10^3^ to 2.5 × 10^8^ copies (R^2^ = 0.991). To determine the ratio of incorporated brucellae/cell, the absolute quantification method was employed, with the implementation of two single-plex reactions, one for the detection of the L-glutamine: 2-deoxy-scylloinosose aminotransferase gene as indicated by the commercial kit and a second one for the wild type ovine genomic DNA for the detection of the endogenous ovine prion protein (ovPrp) single-copy chromosomal gene [11,12] as previously described [9] (range 1–1 × 10^5^ copies, R^2^ = 0.992). The primers for the ovPrp gene (ovPrpF 5′-GCCAAAAACCAACATGAAGCAT-3′ and ovPrpR 5′-TGCTCATGGCACTTCCCAG-3′) yielded a 95-bp amplicon. The standard curves produced were then used to extrapolate the absolute number of brucellae and ovPrp copies per reaction. Ultimately, brucellae per cell were calculated by normalizing the absolute number of brucellae to the ovPrp copies, in order to adjust for equal loading of genomic DNA per reaction. All reactions were conducted in a StepOne Real-Time PCR instrument (Applied Biosystems, Foster City, CA, USA). 

A qualitative assessment of the intracellular and extracellular presence of brucellae was also performed by conventional end-point PCR (all reagents from Invitrogen, Carlsbad, CA, USA) in a total of 50 μL containing 5 μL DNA, 1× Dream Taq buffer, 0.2 mM dNTPs, 0.05 U/μL Dream Taq polymerase and a 1 μM concentration of the BMEII0843f (5′-TTTACACAGGCAATCCAGCA-3′) and BMEII0844r (5′-GCGTCCAGTTGTTGTTGATG-3′) primers as described previously [13], producing a 1071 bp amplicon. The amplification was carried out according to the following conditions: initial denaturation at 95 °C for 7 min and 40 cycles of 95 °C for 1 min, 60 °C for 1 min and 72 °C for 1 min. The amplicons were analyzed by agarose gel electrophoresis.

### 2.4. Statistics

Multiple comparisons were performed using the one-way ANOVA. Values of *p* < 0.05 were considered statistically significant. All results are expressed as means ± standard deviation (SD). 

## 3. Results

### Establishment of the Ovine Macrophage Infection In Vitro Model

**Figure 2:**
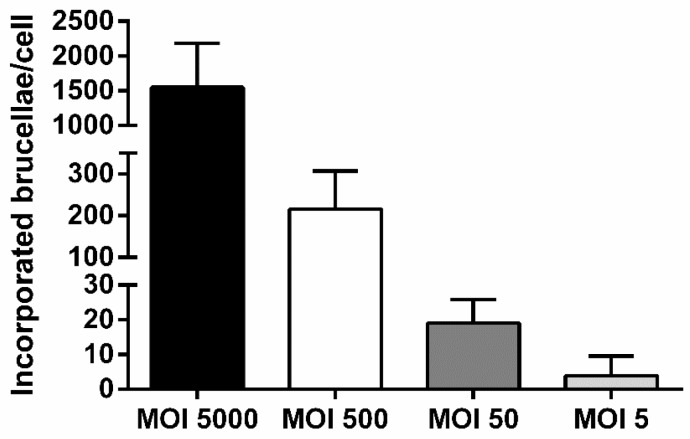
Comparative analysis of incorporated brucellae per cell in ovine macrophages infected with *Brucella* at various multiplicities of infection (MOIs), 2 days post-infection (quantitative Real-Time PCR). Each MOI condition was repeated 6 times (six different experiments on macrophages of 6 different sheep). Data are represented as means ± standard deviation. MOI: multiplicity of infection.

We utilized wild type peripheral blood macrophages from healthy sheep to infect with *Brucella* in serial dilutions that corresponded to an MOI of 5000, 500, 50, or 5 and assess the minimum detection limit of our assay regarding quantification of internalized brucellae by Real-Time PCR. As depicted in Figure 2, the number of intracellular brucellae 48 h post-infection, was proportionally related to the increasing MOIs, since, on average, half of the bacteria indicated per MOI showed successful incorporation into macrophages. The minimum detection limit of our assay was produced at the lowest macrophage: *Brucella* ratio (MOI = 5) and corresponded to 3.97 ± 5.58 incorporated brucellae/cell. As expected, the MOI of 5000 produced the largest numbers of engulfed bacteria into the host cytoplasm (1549.32 ± 635.85 incorporated brucellae/cell). Moreover, the Ct’s produced from the exogenous DNA extraction control fell within the range indicated by the supplier (Ct = 25–31), demonstrating that minimal or no PCR inhibitors were present in our samples. However, it should be noted that the lowest number of total brucellae detectable by Real-Time PCR was approximately 2500 brucellae.

**Figure 3:**
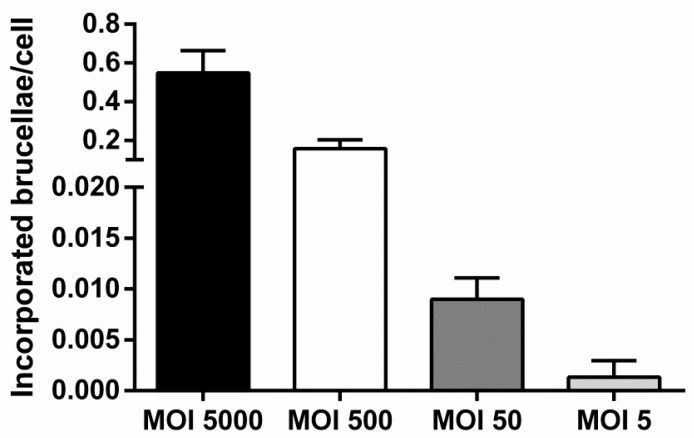
Comparative analysis of incorporated brucellae per cell in ovine macrophages infected with *Brucella* at various MOIs, 2 days post-infection (results from colony-forming unit (CFU) assay from 500 infected macrophages seeded in blood agar dishes). Each MOI condition was repeated 6 times (six different experiments on macrophages of 6 different sheep). Data are represented as means ± standard deviation. MOI: multiplicity of infection.

**Figure 4:**
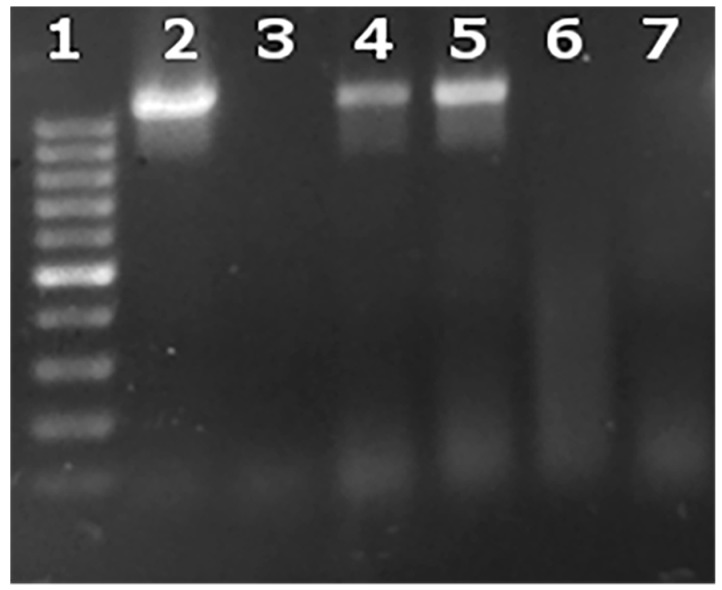
Intracellular and extracellular brucellae from one representative experiment at an MOI = 5000. Lane 1: 100–1000 base pair ladder; Lane 2: culture supernatant from macrophages before addition of lysozyme and antibiotics (Day 1); Lane 3: culture supernatant from macrophages after addition of lysozyme and antibiotics (Day 2); Lane 4: infected macrophages after addition of lysozyme and antibiotics (Day 2); Lane 5: *B. melitensis* 16 M positive control; Lane 6: negative PCR control; Lane 7: uninfected macrophages.

Our results truly reflected intracellular bacteria, since the culture supernatant of the infected cells that was collected 24 h after their exposure to antibiotics and lysozyme and seeded in blood agar dishes, did not yield any colonies at all MOIs based on the CFU assay. Moreover, to verify that all extracellular brucellae were killed before proceeding to DNA extraction, we also washed the infected cells to remove trypsin, after harvesting them from the 24-well plates at Day 2, and utilized the supernatant produced from this final cell wash to seed in blood agar dishes. Once more, no colonies were formed. Additionally, the presence of intracellular live brucellae was verified by lysing 500 infected cells and seeding them in blood agar dishes. After approximately one week, single colonies were formed in all experimental groups that further corroborated our previous observations (Figure 3, Table 1). The aforementioned were also verified with conventional PCR (Figure 4). 

**Figure 5:**
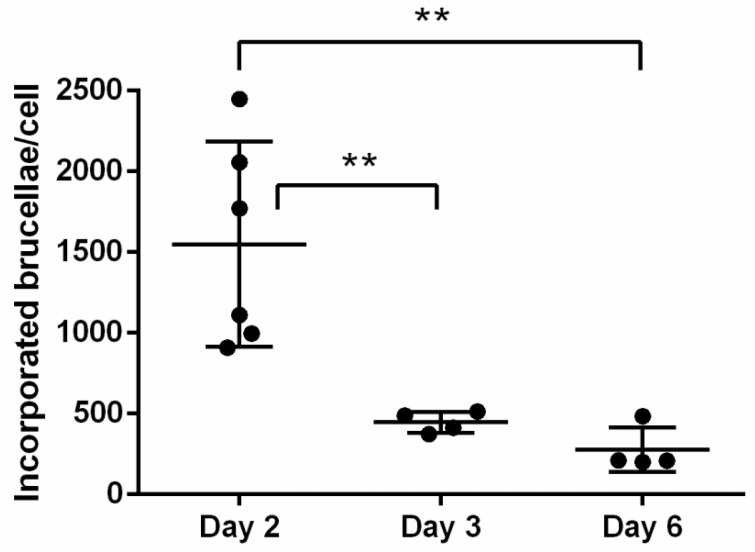
Summative analysis of incorporated brucellae per cell in ovine macrophages infected with *Brucella* at MOI = 5000, on Days 2, 3, and 6 post-infection (quantitative Real-Time PCR). Statistical comparison was performed with the one-way ANOVA and data are expressed as means ± standard deviation. Each MOI condition was repeated 6 times (six different experiments on macrophages of 6 different sheep). ** *p* > 0.005.

Furthermore, we proceeded to culturing the *Brucella*-infected macrophages for up to 6 days, in order to evaluate any potential fluctuations in the number of incorporated brucellae over time, under conditions of heavy bacterial loads produced by the MOI of 5000. Summative analysis of experiments conducted with *Brucella*-infected macrophages at MOI = 5000, demonstrated a gradual drop in the bacterial load associated with duration in culture; from Day 2 to Day 6 post-*Brucella* infection, bacterial numbers were significantly reduced (Day 2: 1549.32 ± 635.85 vs. Day 6: 276.67 ± 138.01, *p* = 0.0013, one-way ANOVA) (Figure 5). This is consistent with a previous report with *B. abortus* in an infected macrophage cell line stating that the number of intracellular brucellae usually peaks at 48 h post-infection and until 96 h the load is gradually dropping [10]. This may be a result of natural intracellular killing of brucellae until they reach residual numbers [6] that basically persist for life, and not of macrophage death, since viability of cells, measured by trypan blue exclusion at all time points, remained >90%. The high variation in incorporated *Brucella* numbers on Day 2 most likely reflects each animal’s sensitivity to infection.

## 4. Discussion

The basic approach for controlling and eradicating brucellosis has always been vaccination of herds, testing animals for infection and sending infected animals to slaughter [3]. These programs have failed in southern Europe and alternatives are necessary to be found [14]. For instance, in mainland Greece, the program has been ongoing since 1978, and it has been failing to decrease the rates of infection under 2%, posing safety concerns towards traditional dairy products, such as feta cheese. In addition, reimbursement costs for seropositive animals, outlines a substantial financial burden not only for the European Union, but also for animal breeders as well [7]. Moreover, the attenuated vaccine used until nowadays, is not entirely safe for veterinarians who administer it and may cause undesired side effects in animals [5]. 

For the first time, we created a cellular model, using macrophages from sheep peripheral blood to simulate infection with *Brucella melitensis*. To the best of our knowledge, all previous studies reporting *B. melitensis* infection models, were based either on cell lines [15,16], or wild type monocytes isolated from peripheral venous blood from healthy volunteers [17]. There are also studies reporting cellular models with wild type sheep macrophages using other *Brucella* species, such as *Brucella ovis* [18]. Here, we exposed wild type ovine macrophages to serial dilutions of the microorganism and subjected the infected cells to Real-Time PCR. Our assay warranted a minimum detectable number of as little as 3.97 internalized brucellae per cell. No PCR inhibitors were detected, that may have led to jeopardizing the integrity of our data. 

Viability of brucellae that were added to macrophages overnight was verified at all MOIs by collecting the culture supernatant, before adding antibiotics and lysozyme to cells, and seeding it, after spinning, to blood agar dishes. Moreover, by lysing 500 infected macrophages after their exposure to antibiotics and lysozyme, we were able to culture the intracellular brucellae in blood agar dishes. It is well-known that isolation and culture of *Brucella* in clinical samples by routine bacteriological culture methods can be particularly challenging due to the slow growth of the microorganism. Since seemingly negative culture dishes are often discarded after a one-week incubation period, diagnosis may be missed altogether. In order to maximize the detection of brucellae, long-term incubation of cultures that may take up to 30 days, as well as blind subculturing have been advised [19]. However, this approach is labor-intensive, expensive and diagnosis is substantially delayed.

Here, we’ve employed different methods, both molecular- and culture-based, to demonstrate the presence of truly engulfed bacteria into the host cytoplasm. Based on our results, the CFU assay generates lower numbers of intracellular brucellae/cell, probably because not all engulfed brucellae can ultimately grow in culture after lysis of macrophages. Another reason for this discrepancy might be that intracellular brucellae are no longer alive but their DNA is still detectable. However, the CFU assay was a lot more sensitive compared to our Real-Time PCR assay since it can detect engulfed brucellae from only 500 infected macrophages with an average copy number of 0.67 as indicated in Table 1 and as indicated in Figure 3 it was able to detect as low as 0.005 or less brucellae/cell, compared to the calculated sensitivity of the Real-Time PCR assay which had a cutoff of 2,500 brucellae in total. These results indicate that in order to reliably diagnose *B. melitensis* by Real-Time PCR, we would need at least 2,500 cells isolated from an infected animal with an average of 1 engulfed *Brucella*/cell. Hence, because the level of sensitivity of the Real-Time PCR was lower compared to the CFU assay, the safe conclusion that can be drawn suggests that the Real-Time PCR could be potentially utilized to detect intracellular brucellae only if there is either a high bacterial load, or if the starting number of cells in the isolated tissue from infected animals is over 2,500. Alternatively, addition of concentration steps in clinical samples would further facilitate detection of residual brucellae by real-time PCR, since culture of *Brucella* in clinical samples by routine bacteriological culture methods can be particularly challenging due to the slow growth of the microorganism. In fact, we are already successfully implementing the method on samples from confirmed *B. melitensis* cases in farms all over Greece (manuscript in preparation). Although culturing is an indispensable method to provide proof of intracellular viable bacteria, we herein provide further insight on the detection of *Brucella* infection with an incremental approach for detecting intracellular brucellae which could accompany the culture-based techniques specifically when results need to be delivered in a short timeline.

Overall, our Real-Time PCR approach could be particularly useful in the detection of persistent *Brucella* infections in animals, where too few bacteria are present in difficult-to-lyse tissues, such as ganglia lymph nodes, and may thus not be easily traced by conventional blood agar culturing methods, given that high cell numbers are obtained from infected animals.

## 5. Conclusions

In summary, the work presented here constitutes a reliable in vitro cell model of *Brucella melitensis* for research purposes that can be utilized to adequately measure the number of engulfed brucellae/cell and provides insights towards future utilization of molecular biology-based methods for detection of *Brucella*.

## Figures and Tables

**Table 1:** Detailed results from all the experiments conducted to determine the number of incorporated brucellae in ovine macrophages infected with *Brucella* at various MOIs, 2 days post-infection either from 500 infected and lysed macrophages seeded in blood agar dishes or from 50,000 infected macrophages analyzed by Real-Time PCR. Each MOI condition was repeated 6 times (six different experiments on macrophages of 6 different sheep). MOI: multiplicity of infection.

		Total Number of Colonies (Agar)	Brucellae/Cell (Real-Time PCR)
MOI 5000	Exp 1	350	2057.46
	Exp 2	232	997.86
	Exp 3	201	909.56
	Exp 4	327	1111.22
	Exp 5	288	1771.21
	Exp 6	249	2448.61
	**Average**	**274.5**	**1549**
	**SD**	**57.44**	**635.8**
MOI 500	Exp 1	103	163.38
	Exp 2	91	131.42
	Exp 3	56	206.07
	Exp 4	83	269.77
	Exp 5	45	150.06
	Exp 6	93	372.37
	**Average**	**78.5**	**215.5**
	**SD**	**22.87**	**91.34**
MOI 50	Exp 1	5	28.94
	Exp 2	6	24.57
	Exp 3	5	15.56
	Exp 4	3	13.32
	Exp 5	4	20.23
	Exp 6	4	11.39
	**Average**	**4.5**	**19.00**
	**SD**	**1.05**	**6.832**
MOI 5	Exp 1	0	0.00
	Exp 2	0	0.00
	Exp 3	0	0.00
	Exp 4	1	6.65
	Exp 5	2	14.00
	Exp 6	1	3.17
	**Average**	**0.67**	**3.97**
	**SD**	**0.82**	**5.58**
